# The incidence of diabetes among the non-diabetic residents in Kawauchi village, Fukushima, who experienced evacuation after the 2011 Fukushima Daiichi nuclear power plant disaster

**DOI:** 10.1186/s12199-020-00852-x

**Published:** 2020-05-08

**Authors:** Yun-Shan Chung, Kouji H. Harada, Keiko Igari, Jinrou Ishizuka, Akio Koizumi

**Affiliations:** 1 Department of Health and Environmental Sciences, Kyoto University Graduate School of Medicine, Kyoto, 606-8501 Japan; 2 Yufune Healthcare Center, Kawauchi Village Office, Fukushima, 979-1202 Japan; 3 Medical Corporation Ishizuka Clinics, Ono Town, Fukushima, 963-3401 Japan; 4 Public Interest Corporation Kyoto Hokenkai, Nakagyo-ku, Kyoto, 616-8141 Japan

**Keywords:** Diabetes, Fukushima Daiichi nuclear power plant accident, Medical health check-up, Disaster, Evacuation

## Abstract

**Objectives:**

After the Fukushima Daiichi nuclear power plant disaster in 2011, residents of Kawauchi village who experienced evacuation had a high risk of suffering from diabetes and metabolic syndrome compared with non-evacuees. In addition to evacuation, lifestyle characteristics can be important factors influencing the development and prognosis of diabetes or glucose tolerance. The current study aimed to evaluate the effects of evacuation (i.e., lifestyle changes) on the incidence of diabetes among the non-diabetic residents of Kawauchi village.

**Methods:**

Design is retrospective cohort study. Annual health examination data of residents of Kawauchi village and control area (Ono town) in Fukushima prefecture from 2008 to 2017, as available from the Japanese National Health Insurance system. Participants were classified into three groups: “Diabetes (DM)” (FBG ≥ 126 mg/dL or HbA1c ≥ 6.5% or hospital visit for DM or usage of diabetic medication), “Borderline DM” (126 mg/dL > FBG ≥ 110 mg/dL or 6.5% > HbA1c ≥ 6.0%, and without hospital visit, and without diabetic medication), and “Normoglycemic” (FBG < 110 mg/dL and HbA1c < 6.0%, and without hospital visit, and without diabetic medication). New onset of diabetes was evaluated and the events or missing data were occurred at health checkup. For this survival analysis, 339 residents in Kawauchi and 598 residents in Ono were included. Average follow-up periods after 2010 were 3.9 years in Kawauchi village and 3.6 years in Ono town.

**Results:**

Compared with the normoglycemic group, incidence of DM was much greater in the borderline DM group, where DM occurred among 38.2% of the group in 2012 and increased to over 60% cumulatively through 2017 in Kawauchi village. DM had a prevalence of 16.3% in 2012, and below 30% in 2017 in borderline DM group of Ono town. Cox proportional hazard regression analysis was applied to non-DM groups at both study sites separately to evaluate the effects of lifestyle changes at each site. While BMI, BMI change, and the lack of regular exercise (HR = 1.29, 1.72, and 5.04, respectively) showed significant associations with the onset of diabetes in Ono town, only BMI and late-night dinner (HR = 1.21 and 4.86, respectively) showed significant associations with diabetes onset in Kawauchi village.

**Conclusions:**

The current results confirmed that diabetes incidence was increased 6 years after the Daiichi nuclear power plant disaster in Kawauchi. We also found changes in lifestyle habits, suggesting that diabetes prevention with promotion of healthy lifestyle behaviors is an urgent priority.

## Introduction

**Fig. 1:**
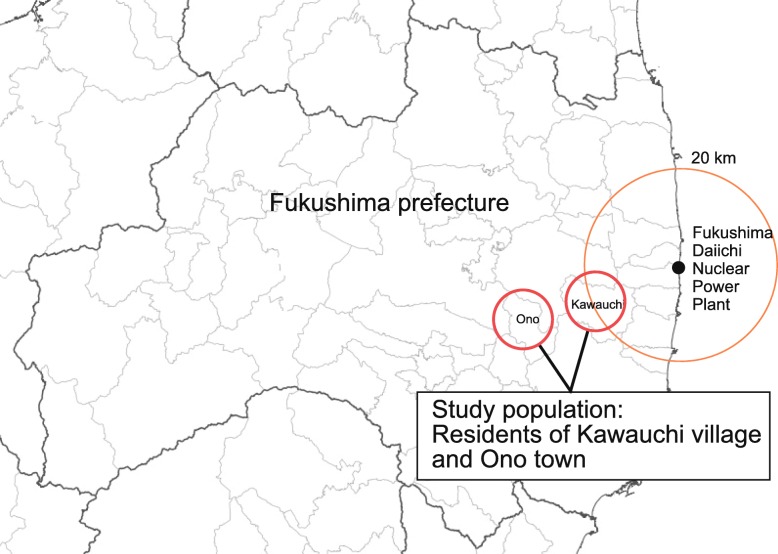
Geographical relationship between the study sites and the Fukushima Daiichi nuclear power plant. The red dotted line shows a 20-km radius of the evacuation zone. Part of Kawauchi village was included in a mandatory evacuation zone. Ono town, used as a control setting in our study, is 5 km from Kawauchi village

On March 11th, 2011, a magnitude 9 earthquake (the Great East Japan Earthquake) inflicted severe damage in the northeastern parts of Japan. The earthquake triggered a powerful tsunami, which resulted in a major nuclear reactor meltdown at the Fukushima Daiichi nuclear power plant (FDNPP) located nearby. The nuclear accident also resulted in an uncertain risk of radioactive release. Immediately after the disaster, around 160,000 residents living in the plant’s vicinity were evacuated [1]. As shown in Fig. 1, Kawauchi village is among the areas located within a 20-km radius of the evacuation order. As a preventive measure, the village’s residents evacuated immediately after the disaster (March 16th, 2011), and only began to return to their homes after April 2012.

Previous studies reported that the prevalence of diabetes significantly increased among evacuees after the Great East Japan Earthquake and the FDNPP disaster. In addition, the increase in HbA1c levels was reported to be significantly greater among evacuees than non-evacuees [2, 3]. Lifestyle habits, such as dietary habits, exercise, sleep, alcohol intake, and smoking are well-known factors affecting glucose metabolism [4]. Since evacuation may greatly affect the lifestyle habits of evacuees, lifestyle changes associated with evacuation may have an impact on evacuees’ glucose metabolism, leading to an increase in the incidence of diabetes. Among those residents, Kawauchi villagers started to return to their homes earlier than others. However, their cross-sectional prevalence of diabetes, metabolic syndrome, dyslipidaemia, hyperuricaemia, and chronic kidney disease increased after disaster and remained high in 2013 [5]. Its temporal change in prevalence was significant, but comparison of the trend should be done with the concurrent control population. Furthermore, subsequent effects of evacuation need to be continuously followed-up after 2013.

This study therefore aimed to evaluate the effects of evacuation (i.e., lifestyle changes) on the incidence of diabetes among the non-diabetic residents after returning to Kawauchi village, compared with the neighboring Ono town, using municipal records of annual health checkup data from 2010 to 2017. Kawauchi villagers lived within 20–30 km radius from FDNPP, and experienced evacuation from March 2011 until at least March 2012. Residents in Ono town lived 30 km away from FDNPP and did not evacuate.

## Methods

### Study design

The current study was a retrospective cohort study. Annual health examination data collected under the National Health Insurance system (NHIS), including anthropometric, biochemical, and lifestyle measurements from 2010, and 2012 to 2017, were obtained from Kawauchi village, and from Ono town as a control, with permission from the local medical facilities. In Ono town, residents under NHIS could select mass examination in community facility or individual examination in designated clinics or hospitals while in Kawauchi village residents took only mass examination. To compare these two municipalities, we limited the participants to only who took mass examination.

Follow-up period was from 2010 to 2017. Endpoint was defined as new-onset of diabetes among non-diabetic group (borderline DM + normoglycemic), normoglycemic group, or borderline DM group as described in the latter section. Censoring of follow up occurred when missing data for diagnosis of diabetes was observed at health checkup.

The current research proposal was approved by the institutional review board of Kyoto University (R0869) and anonymized data were provided with the approval of the mayors of Kawauchi village and Ono town.

### Study population

Retrospective cohorts were established from the annual health examination data of 2010, the year just before the disaster, for both study sites. Individuals were followed up for annual health checkups from 2012 to 2017. If an individual was absent from the annual health check-up, they were eliminated from the follow-up (as censored), and all analyzed participants attended the annual checkups continuously from 2012 onward.

**Fig. 2:**
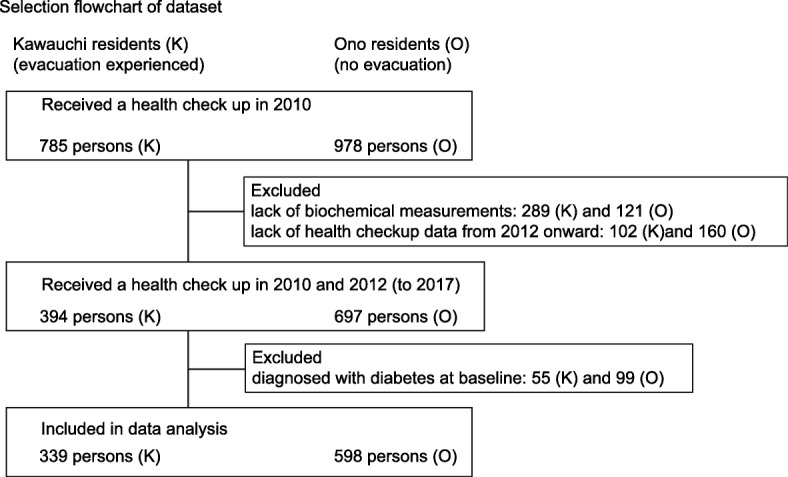
Flow-chart of residents included in this study

In Kawauchi village and Ono town, 785 and 978 residents attended the health checkup in 2010, respectively. Of the residents in Kawauchi village and Ono town, 289 and 121 were excluded because of the lack of biochemical measurements on glucose tolerance, and 102 and 160 were excluded because of the lack of health checkup data from 2012 onward, respectively; 55 and 99 individuals were further eliminated because they were already diagnosed with diabetes, leaving 339 and 598 residents from Kawauchi village and Ono town as baseline cohorts, respectively (Fig. 2).

Local government of Kawauchi village decided evacuation of residents on March 16th, 2011, and made a decision to return to their homes from April 1, 2012. Then, Kawauchi villagers were all classified as experiencing evacuation. While the timing of returning to home differed among Kawauchi residents, further stratification was not conducted. Residents in Ono town were defined as non-evacuees. While voluntary evacuation occurred in Ono, there were only nine persons in official records and no impact on the study [6].

### Biomedical information available from health checkup

Information available from the annual health checkup included age, gender, body weight (BW), body mass index (BMI), systolic blood pressure (sBP), diastolic blood pressure (dBP), fasting plasma glucose (FBG), hemoglobin A1c (HbA1c), high-density lipoprotein cholesterol (HDL-C), low-density lipoprotein cholesterol (LDL-C), triglyceride (TG), γ-glutamyl transpeptidase (γ-GTP), red blood cells (RBC), hemoglobin (Hb), hematocrit (Hct), and uric acid (UA). “BMI change” was calculated by subtracting the 2010 value from the 2012 value.

### Diagnostic criteria of glucose tolerance status

The glucose tolerance status was classified into “diabetes mellitus (DM),” “borderline DM,” and “normoglycemic” based on FBG (mg/dL), HbA1c (%), or medication status (Table 1).

**Table 1:** Definition of glucose tolerance status

Status	Definition
Diabetes mellitus (DM)	FBG ≥ 126 mg/dL or HbA1c ≥ 6.5% or hospital visit for DM or usage of diabetic medication
Borderline DM	126 mg/dL > FBG ≥ 110 mg/dL or 6.5% > HbA1c ≥ 6.0%, and without hospital visit, and without diabetic medication
Normoglycemic	FBG < 110 mg/dL and HbA1c < 6.0%, and without hospital visit, and without diabetic medication

### Health status or lifestyle information available from health checkup

Information available from the questionnaire included the history of pharmaceutical treatment for hypertension, DM, and high cholesterolemia and lifestyle behaviors and attitudes according to the standard questions proposed by the national government for annual health checkups. Lifestyle questions included the following 14 questions with “Yes/No” answers, except for questions on drinking and eating speed: (1) smoking (“Are you currently a habitual smoker?”); (2) frequency of drinking (“How often do you drink? Every day/Sometimes/Rarely/Never); (3) exercise (“Are you having exercise to the point of a light sweat for 30 min or longer at least twice a week for 1 year or more?”); (4) walking (“Do you walk or perform equivalent activity in your daily life for at least 1 hour every day?”); (5) walking speed (“Do you walk fast in comparison to others of your age group?”); (6) restful sleep (“Do you feel rested enough after sleeping overnight”); (7) late-night dinner (“Do you go to bed within 2 h of eating dinner three or more times a week?”); (8) bedtime snacking (“Do you have a late-night snack [in addition to three meals] three or more times a week?”); (9) skipping breakfast (“Do you skip breakfast three or more times a week?”); (10) eating speed (“How fast do you eat compared with other people?” Faster/Normal/Slower); (11) body weight maintenance #1 (“Has your weight increased by 10 kg or more compared with your weight at the age of 20?”; (12) body weight maintenance #2 “Has your weight increased or decreased by 3 kg or more in the past year?”); (13) attitude toward changing lifestyle #1 (“Are you considering or trying to improve your dietary, exercise or other lifestyle habits?”); (14) attitude toward changing lifestyle #2 (“If offered an opportunity to receive guidance under health insurance regarding methods of lifestyle change, would you like to take it?”). Answers for each question were scored as 0 or 1, with 1 representing an undesirable lifestyle (for drinking, a score of 1 was given for “everyday” and a score of 0 was given for any other responses, and for eating speed, a score of 1 was given for “faster” and a score of 0 was given for any other responses). “Lifestyle change score” was calculated by subtracting the sum of the scores of questions (1), (2), (3), (4), (6), (7), (11), and (12) in 2010 from the sum of the scores of these questions in 2012.

### Statistical analysis

For the baseline comparison between Kawauchi village and Ono town residents, Students’ *t* tests were used for continuous variables and chi-square tests were used for categorical variables. The Kaplan-Meier curve and log-rank test were used to compare the time to the event (i.e., the diagnosis of diabetes over time) between two study sites among all non-diabetic residents (borderline DM + normoglycemic) and borderline DM and normoglycemic individuals, separately. Cox proportional hazard regression analysis was further applied to evaluate the hazard ratio for the difference in the incidence of DM between the study sites incorporating selected health checkup variables at baseline as well as the “lifestyle change score” and “BMI change.” We assumed events or missing data occurring at health checkup. We also compared baseline variables between cohort data and original data records of health checkups to examine potential sources of bias. Records with missing data were excluded from Cox regression analyses. The statistical significance level was set at a *p* value less than 0.05. JMP Pro 14 (SAS Institute, Tokyo, Japan) was used for all statistical analysis.

## Results

### Baseline characteristics of the cohorts

Table 2 compares the baseline biomedical and health behavioral characteristics as well as lifestyle change scores and BMI change between the two study sites. Follow up periods in the non-DM group were 1313 and 2173 person-years in Kawauchi and Ono, respectively. Average follow-up periods were 3.9 years in Kawauchi village and 3.6 years in Ono town. Compared with Ono town (control), cohort members in Kawauchi village were 2 years older on average, with a lower proportion of men, and greater BMI, higher sBP, lower dBP, lower FBG, lower blood lipids (triglyceride and LDL-C), less habitual drinking, more habitual walking, greater BMI change, and greater lifestyle change scores. All of these differences were statistically significant.

**Table 2:** Comparison of selected biomedical and health behavioral characteristics and lifestyle change scores between Kawauchi village affected by the 2011 Fukushima Daiichi nuclear plant disaster and Ono town at baseline (2010)

	Kawauchi village (with evacuation)	Ono town (without evacuation)	*p value
**Number of participants**	339	598	
**Demographic/anthropometric variables**			
Age (years)	68.5 (9.8)	66.3 (9.1)	**0.001**
Sex (male/female)	160/179	285/313	**< 0.001**
Body weight (kg)	56.2 (9.9)	56.2 (9.3)	0.997
BMI (kg/m^2^)	23.7 (3.3)	23.2 (2.9)	**0.012**
Systolic blood pressure	134.4 (17.4)	125.7 (14.3)	**< 0.001**
Diastolic blood pressure	75.8 (10.5)	78.1 (9.7)	**< 0.001**
**Biochemical variables**			
Fasting plasma glucose (mg/dL)	92.8 (9.3)	95.9 (9.0)	**< 0.001**
Hemoglobin A1c (%)	5.47 (0.31)	5.45 (0.31)	0.408
Triglyceride (mg/dL)	89.8 (41.6)	104.5 (80.4)	**0.002**
HDL-cholesterol	59.6 (14.4)	58.8 (13.8)	0.397
LDL-cholesterol	114.1 (25.6)	120.4 (28.3)	**0.001**
γ-GTP (IU/L)	28.6 (29.9)	30.8 (34.6)	0.329
Uric acid	5.0 (1.4)		
Hemoglobin (g/dL)	13.7 (1.4)	13.4 (1.2)	0.071
**Lifestyle variables**			
Keeping body weight gain > 10 kg compared with weight at 20 years old (%)	33.5%	30.1%	0.344
Keeping body weight gain or loss within 3 kg compared with weight in the last year (%)	25.2%	20.4%	0.130
Currently smoking (%)	11.8%	13.9%	0.362
Drinking everyday (%)	24.2%	27.9%	**< 0.001**
Regular exercise: ≥ 30 min/time and ≥ 2 times/week for 1 year or more (%)	37.9%	35.2%	0.473
Walking at least 1 h/day (%)	48.4%	40.5%	**0.036**
Restful sleep: getting enough rest by sleep (%)	71.9%	77.5%	0.089
Late-night dinners: ≥ 3 times/week (%)	21.8%	27.4%	0.094
Lifestyle change score (*n* = 216 for Kawauchi village and *n* = 456 for Ono village)	0.21 (1.34)	−0.04 (1.27)	**0.016**
Difference of BMI	0.68 (1.42)	0.21 (0.79)	**< 0.001**

Data are presented as mean (standard deviation) and the percentage of lifestyle represents number of “yes”/total responses. Lifestyle change scores were calculated using the sum of the scores for lifestyle questions in this table in 2012 subtracted by the summed scores of these questions in 2010

*BMI* body mass index, *HDL/LDL* high/low-density lipoprotein, *γ-GTP* γ-glutamyl transpeptidase

### Survival analysis

**Fig. 3:**
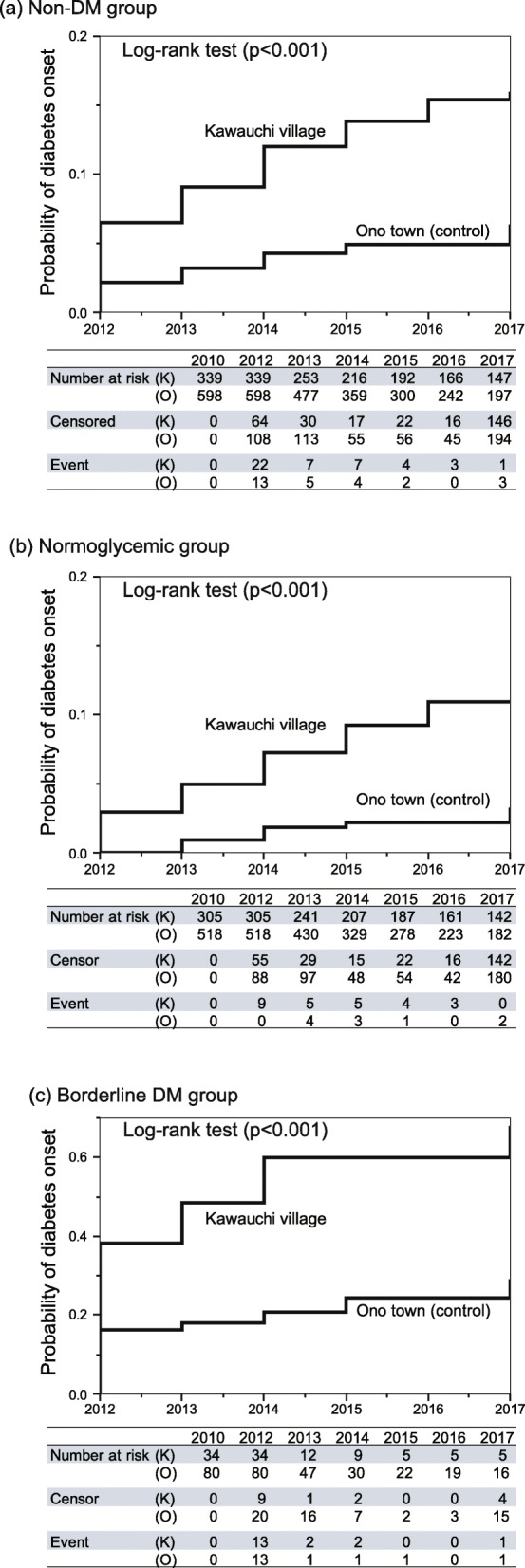
Kaplan-Meier curves and the results of log-rank tests comparing the occurrence of diabetes mellitus (DM) between Kawauchi village and Ono town: (**a**) Non-diabetic group (borderline DM + normoglycemic), (**b**) normoglycemic group, and (**c**) Borderline DM

Figure 3 shows the Kaplan-Meier curves and the results of the log-rank tests to compare the occurrence of DM over time between two study sites: Fig. 3a for all non-diabetic residents (borderline DM + normoglycemic), Fig. 3b for normoglycemic residents, and Fig. 3c for borderline DM residents. Logarithm cumulative hazard plots between two sites are fairly parallel for those three categories (Figure S1), and proportional hazard of evacuation effect was assumed in Cox regression analysis. Significant differences were detected between the two villages in all groups by the log-rank tests. Compared with the normoglycemic group, the incidence of DM was much greater in the borderline DM group, where DM occurred among 38.2% of the members in 2012, and increased to over 60% cumulatively in 2017 in Kawauchi village, while DM occurred only in 16.3% of residents in 2012 and less than 30% of residents in 2017 in Ono town.

To compare the difference in the incidence of DM between two study sites, Cox proportional hazard regression analysis was performed, adjusting for selected health checkup variables at the baseline (2010) as well as BMI change and the lifestyle change score that reflect changes in lifestyle from 2010 to 2012. Selection of the variables was based on previous studies [2, 3] in this area and epidemiological importance. In this analysis, the total number of participants decreased from 937 (Table 2) to 667 because lifestyle scores or BMI changes could not be calculated for 267 residents because of the lack of data in 2012. As shown in Table 3, the experience of evacuation (Kawauchi village), BMI and late-night dinner were significantly associated with greater hazard ratio (HR) for developing DM in the non-DM group (HR = 2.39, 1.17, and 2.90, respectively), while significantly elevated HRs were detected for evacuation, male gender, BMI, and late-night dinner (HR = 5.09, 2.93, 1.22, and 5.05, respectively) in the normoglycemic group, while only BMI (HR = 1.18) was significantly associated in the borderline DM group. The incidence rates of DM in Kawauchi and Ono were 3.4 and 1.2 per 100 person-years in non-DM group; 2.1 and 0.5 in normoglycemic group; 25.7 and 7.9 in the borderline group, respectively. The incidence of DM among non-DM group in Ono town was comparable to that of epidemiological studies of Japanese individuals in 2000 or later (1.34 per 100 person-years [95% CI, 10.4–17.1]) [4].

**Table 3:** Cox proportional hazard regression analyses to estimate the effect of evacuation on diabetes mellitus incidence, adjusting for selected demographic and anthropometric and health behavior variables, as well as lifestyle change scores and BMI changes using the annual health check-up data of Kawauchi village (with evacuation) and Ono town (without evacuation)

Correlates	Total	Normoglycemic	Borderline diabetes
	(*n* = 667)	(*n* = 589)	(*n* = 78)
Evacuation (Yes = 1; No = 0)	2.39 [1.28–4.50]	5.09 [2.03–14.5]	2.94 [0.89–10.1]
Age	1.02 [0.97–1.07]	0.98 [0.92–1.03]	0.96 [0.88–1.05]
Gender	1.91 [0.95–3.82]	2.93 [1.07–8.23]	1.21 [0.40–3.51]
BMI	1.19 [1.08–1.30]	1.22 [1.06–1.40]	1.18 [1.03–1.37]
dBMI	0.98 [0.79–1.22]	0.96 [0.72–1.29]	1.54 [0.86–2.80]
Keeping body weight gain > 10 kg compared with weight at 20 years old (%) (Yes = 1, No = 0)	1.20 [0.58–2.49]	2.72 [0.89–8.32]	0.80 [0.29–2.31]
Keeping body weight gain or loss ≥ 3 kg compared with weight in the last year (%) (Yes = 1, No = 0)	0.85 [0.42–1.70]	0.84 [0.32–2.25]	0.84 [0.30–2.49]
Currently smoking (%) (Yes = 1, No = 0)	0.75 [0.34–1.65]	0.82 [0.28–2.42]	0.44 [0.09–2.12]
Drinking everyday (%) (Yes = 1, No = 0)	1.05 [0.51–2.15]	1.07 [0.40–2.87]	1.05 [0.29–3.79]
Regular exercise: ≥ 30 min/time and ≥ 2 times/week for 1 year or more (%) (Yes = 0, No = 1)	1.53 [0.75–3.10]	1.43 [0.54–3.77]	2.26 [0.64–7.92]
Walking at least 1 h/day (%) (Yes = 0, No = 1)	0.67 [0.32–1.38]	1.20 [0.46–3.13]	0.57 [0.15–2.09]
Restful sleep: getting enough rest by sleep (%) (Yes = 0, No = 1)	0.65 [0.35–1.24]	0.77 [0.32–1.84]	0.30 [0.09–1.08]
Late-night dinners: ≥ 3 times/week (%) (Yes = 1, No = 0)	2.90 [1.14–7.38]	5.05 [1.04–24.5]	0.78 [0.21–3.26]
Lifestyle change score	1.03 [0.80–1.33]	1.28 [0.90–1.81]	0.79 [0.50–1.26]

Data are presented as hazard ratio [95% confidence interval]

Lifestyle change scores were calculated by subtracting the sum of the scores of lifestyle questions in the table in 2010 from the sum of scores in 2012. BMI change was calculated by subtracting the value in 2010 from the value in 2012

*BMI* body mass index

Cox proportional hazard regression analysis was further applied to non-DM groups at two study sites separately to evaluate the effect of lifestyle changes in each site. While BMI, BMI change, and the lack of regular exercise (HR = 1.29, 1.72 and 5.04, respectively) exhibited a significant association with the onset of diabetes in Ono Town, only BMI and late-night dinner (HR = 1.21 and 4.86, respectively) showed significant associations in Kawauchi village (Table 4).

**Table 4:** Cox proportional hazard regression analyses to estimate the effect of lifestyle and BMI changes on the incidence of diabetes mellitus (DM) among non-DM group in Kawauchi village (with evacuation) and Ono town (without evacuation)

Correlates	Kawauchi Village	Ono Town
	(*n* = 210)	(*n* = 457)
Age	1.04 [0.98–1.11]	1.00 [0.93–1.07]
Gender	1.89 [0.75–4.70]	2.85 [0.88–9.71]
BMI	1.21 [1.06–1.37]	1.29 [1.10–1.51]
dBMI	0.92 [0.72–1.20]	1.72 [1.03–2.86]
Keeping body weight gain > 10 kg compared with weight at 20 years old (%) (Yes = 1, No = 0)	1.70 [0.67–4.71]	1.03 [0.31–3.42]
Keeping body weight gain or loss ≥ 3 kg compared with weight in the last year (%) (Yes = 1, No = 0)	1.09 [0.44–3.05]	0.37 [0.14–1.00]
Currently smoking (%) (Yes = 1, No = 0)	0.94 [0.31–3.26]	0.45 [0.15–1.40]
Drinking everyday (%) (Yes = 1, No = 0)	1.15 [0.45–3.38]	1.01 [0.33–3.10]
Regular exercise: ≥ 30 min/time and ≥ 2 times/week for 1 year or more (%) (Yes = 0, No = 1)	1.00 [0.39–2.48]	5.04 [1.48–17.2]
Walking at least 1 h/day (%) (Yes = 0, No = 1)	0.66 [0.26–1.61]	0.42 [0.12–1.40]
Restful sleep: getting enough rest by sleep (%) (Yes = 0, No = 1)	0.89 [0.40–2.21]	0.32 [0.12–0.90]
Late-night dinners: ≥ 3 times/week (%) (Yes = 1, No = 0)	4.86 [1.26–33.4]	1.79 [0.53–6.03]
Lifestyle change score	1.02 [0.74–1.41]	0.99 [0.65–1.50]

Data are presented as hazard ratio [95% Confidence Interval]. Lifestyle change score was calculated by subtracting the sum of the scores of lifestyle questions in this table in 2010 from the sum of the scores in 2012. BMI change was calculated by subtracting the value in 2010 from the value in 2012

*BMI* body mass index

We compared the total population and the cohort population in Kawauchi village and Ono town in 2010, 2012 to 2017 (Additional file 1 (Tables S1-S2) provides the full list of results). There was no significant difference in Kawauchi village. However, ages during 2014 to 2017 were significant differences between the total and cohort population in Ono town.

## Discussion

DM is a serious health concern, as one of the major causes of cardiovascular morbidity and mortality [7]. Control of type II DM is particularly important because it is caused by lifestyle factors such as obesity [8–10], lack of exercise [11], stress [12], quality or quantity of sleep [13–15] of and other lifestyle-related factors [16, 17], and is therefore preventable. Given the deep influence of the disaster on wide-ranging aspects of lifestyle among the victims, prevention and control of DM is one of the most important medical problems in populations affected by the disaster.

Because of younger people skipping health checkups, the age of the Ono cohort was higher than that of the total population. Although the Ono cohort had an older population, there was no significant difference in blood glucose or HbA1c compared with the total population. In addition, the Ono cohort may have been healthier than the total population, and there was unlikely to have been overestimation in comparison with the Kawauchi cohort.

The current results indicated that incidence of DM was greater in the village that experienced evacuation because of the 2011 FDNPP disaster compared with the town that did not experience evacuation. Our study further indicated that the incidence of DM was much greater among the borderline DM group compared with the normoglycemic group, suggesting that people with borderline DM should be the priority group for DM prevention after the disaster. The current results were consistent with a previous study [2, 3] conducted in the same area (Fukushima prefecture), which showed that an increase in the incidence of DM and borderline DM was associated with the experience of evacuation after the disaster. In addition to the previous study in Kawauchi village [5], the effects of evacuation on DM continued until 2017 with comparison to non-evacuated area. The current study is the first to report the magnitude and speed of developing DM over time in a normoglycemic group and a borderline DM group separately.

The current study, however, failed to show clear evidence of the influence of lifestyle changes associated with the disaster. Although lifestyle change scores were significantly greater in the village that experienced evacuation (Kawauchi village) compared with the control town (Ono town), it did not show a significant association with the occurrence of DM. Although no definitive conclusions can be drawn, this may be due to the limited ability of the lifestyle questions in the annual health checkup to detect the situation of people affected by the disaster, particularly the magnitude and variety of stress they have experienced. Stress could have influenced the incidence of DM in our study population since increased anxiety and stress due to reduced outdoor activity to avoid exposure to radiation has been reported among the victims of the same area [3]. Unexpectedly, BMI changes and the lack of regular exercise were associated with the incidence of DM in the control town (Ono town) but not in the village that experienced evacuation (Kawauchi village). This may suggest that the impact of stress from the disaster was too strong and masked the effects of other moderate lifestyle alterations.

Whatever the mechanism of the increased incidence of DM after the disaster, immediate introduction of a DM prevention program is critical, as the illness could be developed among 10% of normoglycemic and as high as 60% of borderline DM groups within 5 years of the disaster.

The effects of evacuation should be further investigated in other municipalities.

## Limitations

The current study involved several important limitations that should be considered. First, there may have been a selection bias in the samples because many residents did not attend the examination and dropped out of the cohort. Individuals with DM may have been less likely to attend the annual health checkup. This could lead to underestimation of the event rates due to a sampling bias for healthy individuals. Nonetheless, the rate in the control area was similar to the rate reported in a meta-analysis of Japanese studies [4]. Second, lifestyle information may not have been accurate or comprehensive because the questionnaire used in the annual health checkup was not designed to detect the specific situation of people affected by the disaster, especially in terms of stress. Third, evacuation status may differ among Kawauchi villagers because not all residents returned in April 2012. Hence, the incidence of diabetes might occur more among continued evacuees. On the other hand, some residents in Ono town might voluntarily evacuate. Even if some of the participants experienced voluntary evacuation, it only reduces the hazard ratio between the two regions, and does not cause overestimation of evacuation effects. Fourth, baseline characteristics of participants were not identical as shown in Table 2 while this is not surprising in the observational study. Participants from Kawauchi had higher age, and might be more susceptible to DM than those from Ono. However, the cohort in Kawauchi showed a lower proportion of men, lower FBG, lower blood lipids (triglyceride and LDL-C), which would lower DM risk. They indicated statistical significance (*p* < 0.05), and nonetheless, the actual difference is not clinically significant. Possible confounding factors were incorporated in Cox regression analyses and hazard ratio of evacuation was significantly higher than other factors. Therefore, the effects of evacuation are less biased in this study. Fifth, in this study, onset of diabetes was determined by a single assessment of fasting blood glucose and HbA1c. It is not identical with the clinical diagnosis of diabetes and may cause discrepancy from the situation of clinical diabetes. However, in epidemiological study, the guideline of Japan Diabetes Society does not necessarily require oral glucose tolerance test [18]. At least, even in this definition, the trend of incidence warrants further study and intervention for the residents. Finally, residential information after the disaster was not available for the participants. It was not therefore clear whether the participants of Kawauchi village stayed in their original home or temporary housing, or whether they lived together with their family or alone. It is possible that people living away from their homes were under more stress, and at an increased risk of developing DM. This association, however, could not be evaluated in the current study.

## Conclusions

After the FDNPP disaster, the incidence of DM in Kawauchi residents was increased in both borderline DM and normoglycemic groups, with a much greater incidence among people with borderline DM. Although the effect of lifestyle was not clearly detected in the village that experienced evacuation, this may be due to the limited ability of the questionnaire to accurately or comprehensively detect the situation of the affected population, or could have been due to the overwhelming impact of the disaster masking the effects of other moderate lifestyle alterations. Regardless of the mechanism, the current study revealed the magnitude and the speed of developing DM over time among the victims of the disaster, suggesting the importance of immediate introduction of DM prevention programs in affected areas.

## Supplementary information

**Additional file 1: Table S1.** Comparison of selected biomedical characteristics between total population and cohort population in Kawauchi Village in 2010, 2012 to 2017. **Table S2.** Comparison of selected biomedical characteristics between total population and cohort population in Ono town in 2010, 2012 to 2017.

**Additional file 2: Figure S1.** Logarithm cumulative hazard plots comparing the occurrence of diabetes mellitus (DM) between Kawauchi village and Ono town: (a) Non-diabetic group (borderline DM + normoglycemic), (b) normoglycemic group, and (c) Borderline DM.

## Data Availability

The dataset supporting the conclusions of this article is included within the article.
