# Supplementary material for: The incidence of diabetes among the non-diabetic residents in Kawauchi village, Fukushima, who experienced evacuation after the 2011 Fukushima Daiichi nuclear power plant disaster
Source: Environ Health Prev Med. 2020 May 8;25:13. doi: 10.1186/s12199-020-00852-x (PMC7210664; doi:10.1186/s12199-020-00852-x)
Supplement: Supplementary file 1 — Additional file 1: Table S1. Comparison of selected biomedical characteristics between total population and cohort population in Kawauchi Village in 2010, 2012 to 2017. Table S2. Comparison of selected biomedical characteristics between total population and cohort population in Ono town in 2010, 2012 to 2017. [file 12199_2020_852_MOESM1_ESM.docx]

| **Table S1** Comparison of selected biomedical characteristics between total population and cohort population in Kawauchi Village in 2010, 2012 to 2017. | | | | | | | | |
| --- | --- | --- | --- | --- | --- | --- | --- | --- |
|  |  | 2010 | 2012 | 2013 | 2014 | 2015 | 2016 | 2017 |
| **Number of participants** | [T] | 599 | 519 | 473 | 450 | 478 | 482 | 458 |
|  | [C] | 599 | 461 | 363 | 317 | 295 | 261 | 236 |
| **Demographic/anthropometric variables** | |  |  |  |  |  |  |  |
| Age (years) | [T] | 66.7 (10.32) | 69.3 (10.26) | 69.9 (10.09) | 70.4 (9.64) | 70.8 (9.61) | 71.6(9.52) | 72.4(9.37) |
|  | [C] | 66.7 (10.32) | 69.4 (10.01) | 70.3 (9.76) | 71.1 (9.34) | 71.6 (9.07) | 72.2(8.95) | 73.0(8.83) |
| Sex (male/female) | [T] | 242/357 | 220/299 | 196/277 | 183/267 | 197/281 | 202/280 | 186/272 |
|  | [C] | 242/357 | 198/263 | 154/209 | 136/181 | 127/168 | 116/145 | 106/130 |
| Body weight (kg) | [T] | 56.6 (9.75) | 57.7 (10.91) | 57.4 (11.07) | 57.2 (11.53) | 57.60 (10.99) | 57.2(10.38) | 57.4(10.90) |
|  | [C] | 56.6 (9.75) | 57.9 (10.94) | 57.6 (11.12) | 57.7 (11.60) | 57.84 (11.50) | 57.7(10.39) | 57.52(10.72) |
| BMI (kg/m^2^) | [T] | 23.8 (3.23) | 24.5 (3.66) | 24.2 (3.66) | 24.3 (3.81) | 24.4 (3.72) | 24.4(3.53) | 24.2(3.68) |
|  | [C] | 23.8 (3.23) | 24.5 (3.67) | 24.2 (3.68) | 24.4 (3.92) | 24.4 (3.89) | 24.4(3.53) | 24.0(3.49) |
| Systolic pressure | [T] | 133.9 (17.09) | 127.1 (14.34) | 126.4 (14.86) | 124.7 (12.40) | 128.0 (14.19) | 131.1(16.91) | 134.0(16.71) |
|  | [C] | 133.9 (17.09) | 127.1 (14.55) | 126.2 (14.95) | 124.6 (12.34) | 128.2 (13.81) | 130.6(15.83) | 134.0(17.27) |
| Diastolic pressure | [T] | 76 (10.41) | 74.2 (9.20) | 71 (9.74) | 72.3 (8.13) | 73.0 (10.07) | 73.0(10.41) | 72.5(11.22) |
|  | [C] | 76 (10.41) | 74.1 (9.35) | 70.7 (9.61) | 72.2 (8.12) | 72.3 (10.46) | 72.5(10.03) | 72.7(11.08) |
| **Biochemical variables** |  |  |  |  |  |  |  |  |
| Fasting plasma glucose (mg/dL) | [T] | 96.4 (16.5) | 100.9 (20.15) | 102.9 (18.72) | 102.6 (18.61) | 103.0 (17.33) | 102.5(16.85) | 106.0(19.27) |
|  | [C] | 96.4 (16.5) | 101.2 (20.81) | 103.3 (17.59) | 103.2 (17.94) | 103.5 (18.52) | 103.2(17.12) | 106.0(17.22) |
| Hemoglobin A1c (%) | [T] | 5.6 (0.61) | 5.6 (0.68) | 5.8 (0.72) | 5.7 (0.58) | 5.8 (0.59) | 5.8(0.55) | 5.8(0.56) |
|  | [C] | 5.6 (0.61) | 5.7 (0.71) | 5.8 (0.67) | 5.8 (0.58) | 5.8 (0.58) | 5.8(0.54) | 5.8(0.55) |
| Triglyceride (mg/dL) | [T] | 94.9 (51.42) | 101.1 (49.90) | 107.1 (68.9) | 102.4 (50.78) | 114.8 (64.58) | 114.5(62.80) | 106.5(52.77) |
|  | [C] | 94.9 (51.42) | 98.3 (45.54) | 105 (66.83) | 99.8 (48.65) | 108.3 (55.10) | 109.2(55.55) | 100.6(43.32) |
| HDL-cholesterol | [T] | 58.3 (14.09) | 56.7 (14.24) | 57.2 (13.61) | 58.4 (13.92) | 58.7 (13.74) | 59.5(13.56) | 61.0(14.03) |
|  | [C] | 58.3 (14.09) | 57.2 (14.30) | 57.6 (13.32) | 59.2 (14.16) | 59.7 (13.81) | 60.3(13.39) | 61.6(13.64) |
| LDL-cholesterol | [T] | 115.3 (26.7) | 116.3 (29.57) | 118.7 (30.14) | 116.6 (29.49) | 116.2 (31.19) | 114.6(30.31) | 113.1(29.47) |
|  | [C] | 115.3 (26.7) | 116.7 (28.94) | 117.4 (28.89) | 114.7 (29.20) | 114.0 (27.84) | 112.8(28.38) | 110.7(27.75) |
| γ-GTP (IU/L) | [T] | 28.5 (27.52) | 33.8 (34.14) | 36.1 (54.36) | 32.5 (34.47) | 36.6 (44.90) | 34.5(37.20) | 31.9(27.16) |
|  | [C] | 28.5 (27.52) | 32.8 (30.18) | 36 (57.73) | 31.8 (27.56) | 33.4 (30.43) | 31.8(23.55) | 31.2(24.48) |
| Uric acid | [T] | 5.0 (1.34) | 5.28 (1.41) | 5.2 (1.34) | 5.2 (1.34) | 5.3 (1.34) | 5.3(1.35) | 5.3(1.34) |
|  | [C] | 5.0 (1.34) | 5.3 (1.39) | 5.3 (1.31) | 5.3 (1.32) | 5.3 (1.29) | 5.4(1.28) | 5.3(1.32) |
| Hemoglobin (g/dL) | [T] | 13.7 (1.37) | 13.8 (1.48) | 13.9 (1.42) | 14.0 (1.45) | 14.0 (1.45) | 13.9(1.42) | 14.1(1.46) |
|  | [C] | 13.7 (1.37) | 13.8 (1.43) | 13.4 (1.40) | 14.1 (1.40) | 14.0 (1.40) | 14.0(1.45) | 14.2(1.48) |
| T, total participants of the annual health examination; C, cohort defined in this study; Data are presented as mean (standard deviation). BMI, body mass index; HDL/LDL, high/low-density lipoprotein; γ-GTP, γ-glutamyl transpeptidase. Students’ t-test was used for continuous variables and chi-square test for categorical variables. There were no significant differences between cohort and total population. | | | | | | | | |
|  |  |  |  |  |  |  |  |  |
|  |  |  |  |  |  |  |  |  |
|  |  |  |  |  |  |  |  |  |

| **Table S2** Comparison of selected biomedical characteristics between total population and cohort population in Ono town in 2010, 2012 to 2017. | | | | | | | | |
| --- | --- | --- | --- | --- | --- | --- | --- | --- |
|  |  | 2010 | 2012 | 2013 | 2014 | 2015 | 2016 | 2017 |
| **Number of participants** | [T] | 857 | 792 | 760 | 683 | 664 | 596 | 542 |
|  | [C] | 857 | 680 | 552 | 446 | 380 | 314 | 257 |
| **Demographic/anthropometric variables** | |  |  |  |  |  |  |  |
| Age (years) | [T] | 66.4 (9.38) | 68.4 (9.18) | 68.9 (9.24) | 69.6 (9.02)* | 70.3 (8.91)* | 71.4(8.68)* | 72.5(8.48)* |
|  | [C] | 66.4 (9.38) | 68.6 (8.99) | 69.7 (8.59) | 70.8 (8.26)* | 71.7 (8.13)* | 73.1(7.78)* | 74.5(7.60)* |
| Sex (Male/Female) | [T] | 423/434 | 374/418 | 353/407 | 331/352 | 299/365 | 278/318 | 261/281 |
|  | [C] | 423/434 | 331/349 | 262/290 | 217/229 | 182/198 | 150/164 | 124/133 |
| Body weight (kg) | [T] | 57.1 (10.04) | 56.8 (9.92) | 57.0 (10.37) | 57.1 (10.04) | 56.4 (9.84) | 56.5(9.95) | 56.0(9.98) |
|  | [C] | 57.1 (10.04) | 56.7 (9.61) | 56.3 (9.58) | 56.1 (9.44) | 56.1 (9.43) | 56.0(9.36) | 55.9(9.33) |
| BMI (kg/m^2^) | [T] | 23.4 (3.15) | 23.6 (3.22) | 23.7 (3.28) | 23.7 (3.15) | 23.5 (3.18) | 23.6(3.15) | 23.4(3.15) |
|  | [C] | 23.4 (3.15) | 23.6 (3.13) | 23.5 (3.09) | 23.4 (3.07) | 23.5 (3.06) | 23.5(3.11) | 23.5(3.12) |
| Systolic pressure | [T] | 126.6 (14.54) | 126.8 (15.77) | 123.5 (13.91) | 127.0 (16.13) | 125.6 (15.94) | 134.2(18.37) | 131.1(17.54) |
|  | [C] | 126.6 (14.54) | 126.5 (15.59) | 122.9 (13.51) | 126.7 (15.73) | 125.6 (15.49) | 134.8(18.21) | 132.4(17.52) |
| Diastolic pressure | [T] | 78.4 (9.63) | 73.0 (9.63) | 73.0 (9.10) | 10.45 (71.62) | 71.6 (11.08) | 76.1(12.00) | 72.6(11.64) |
|  | [C] | 78.4 (9.63) | 72.8 (9.67) | 72.4 (9.00) | 70.9 (10.13) | 71.1 (10.92) | 76.1(12.17) | 72.2(11.44) |
| **Biochemical variables** |  |  |  |  |  |  |  |  |
| Fasting plasma glucose (mg/dL) | [T] | 100.8 (19.11) | 99.7 (18.23) | 101.7 (22.59) | 102.3 (17.95) | 101.9 (16.10) | 103.5(18.77) | 102.9(22.21) |
|  | [C] | 100.8 (19.11) | 99.7 (18.12) | 101.2 (20.27) | 102.2 (18.13) | 102.6 (16.53) | 103.9(17.12) | 104.4(19.12) |
| Hemoglobin A1c (%) | [T] | 5.6 (0.64) | 5.6 (0.56) | 5.7 (0.65) | 5.7 (0.64) | 5.7 (0.57) | 5.8(0.61) | 5.80(0.68) |
|  | [C] | 5.6 (0.64) | 5.6 (0.55) | 5.7 (0.60) | 5.7 (0.59) | 5.8 (0.58) | 5.8(0.57) | 5.8(0.54) |
| Triglyceride (mg/dL) | [T] | 107.7 (81.80) | 109.2 (75.36) | 116.4 (74.73) | 115.0 (74.96) | 109.3 (72.16) | 109.4(57.96) | 111.7(57.37) |
|  | [C] | 107.7 (81.80) | 108.6 (74.19) | 111.6 (68.58) | 109.1 (73.70) | 104.0 (52.08) | 106.0(53.18) | 108.8(53.92) |
| HDL-cholesterol | [T] | 58.1 (13.54) | 57.2 (13.68) | 57.6 (14.29) | 57.2 (13.89) | 58.2 (14.53) | 59.8(14.96) | 59.76(14.34) |
|  | [C] | 58.1 (13.54) | 56.9 (13.60) | 57.8 (14.60) | 58.2 (14.58) | 58.1 (14.98) | 58.9(15.00) | 58.6(14.54) |
| LDL-cholesterol | [T] | 121.6 (29.04) | 119.6 (29.67) | 122.2 (30.54) | 122.0 (27.82) | 124.6 (30.65) | 122.4(30.22) | 120.8(30.63) |
|  | [C] | 121.6 (29.04) | 119.2 (29.46) | 122.0 (29.76) | 120.8 (27.90) | 123.2 (29.02) | 120.9(28.85) | 119.4(29.78) |
| γ-GTP (IU/L) | [T] | 33.1 (45.51) | 32.9 (46.10) | 33.1 (39.23) | 30.8 (31.90) | 30.3 (26.67) | 31.0(33.72) | 30.4(28.48) |
|  | [C] | 33.1 (45.51) | 32.9 (47.35) | 31.8 (38.50) | 29.9 (31.50) | 29.1 (24.74) | 29.7(29.15) | 29.8(30.17) |
| Uric acid | [T] | - | 5.2 (1.45) | 5.3 (1.39) | 5.2 (1.32) | 5.3 (1.44) | 5.2(1.29) | 5.3(1.30) |
|  | [C] | - | 5.2 (1.38) | 5.3 (1.36) | 5.2 (1.25) | 5.3 (1.47) | 5.2(1.30) | 5.3(1.31) |
| Hemoglobin (g/dL) | [T] | 13.5 (1.36) | 13.6 (1.41) | 13.7 (1.43) | 13.9 (1.46) | 13.5 (1.42) | 13.8(1.47) | 13.7(1.44) |
|  | [C] | 13.5 (1.36) | 13.6 (1.43) | 13.7 (1.40) | 13.9 (1.42) | 13.5 (1.45) | 13.7(1.53) | 13.7(1.53) |
| T, total participants of the annual health examination; C, cohort defined in this study; Data are presented as mean (standard deviation). BMI, body mass index; HDL/LDL, high/low-density lipoprotein; γ-GTP, γ-glutamyl transpeptidase. Students’ t-test was used for continuous variables and chi-square test for categorical variables. “*”, p-value < 0.05 | | | | | | | | |
|  |  |  |  |  |  |  |  |  |
|  |  |  |  |  |  |  |  |  |
|  |  |  |  |  |  |  |  |  |
